# Selection and recruitment of pre-registration occupational therapy students in the United Kingdom: Exploring entry criteria across education providers

**DOI:** 10.1177/03080226221148412

**Published:** 2023-01-11

**Authors:** Sarah Louise McGinley, Sukhbinder Hamilton, Alexander Bradley

**Affiliations:** 1 Occupational Therapy, School of Health Sciences, University of Southampton, Southampton, UK; 2 University of Portsmouth, Portsmouth, UK

**Keywords:** Admission, occupational therapy, inclusive practices, widening, participation

## Abstract

**Introduction:**

Since widening access to higher education became a United Kingdom (UK) governmental priority in the 1990’s, occupational therapy has made little progress in diversifying student and workforce populations that mirror increasingly diverse service user populations. This research aims to, for the first time, map entry criteria across UK pre-registration programmes, while considering fair access and exploring who might be missing at the point of enquiry and entry.

**Method:**

A cross-sectional quantitative content analysis was conducted of all UK university websites, identifying programme type, academic, professional and alternative entry criteria for 2021/2022.

**Findings:**

Five entry routes via undergraduate and post-graduate programmes (*n* = 73) offer limited part-time opportunities (n = 11). Visible academic entry criteria appear weighted towards ‘traditional’ qualifications, while assessment of professional skills at application and selection is explicit for over 75% of programmes. 86% (n = 63) utilise interviews at selection, with 33% (n = 24) not publicly acknowledging alternative access routes into the profession.

**Conclusion:**

If the profession is to avoid continued stagnation in diversity amongst student populations and successfully reflect service user diversity in the workforce, it is essential UK universities increase parity across academic entry criteria, ensure the visibility of acceptable skills for alternative access and substantially improve flexibility for part-time study.

## Introduction

Widening access and participation to Higher Education (HE) in the United Kingdom (UK) became a key governmental priority in the 1990’s, coinciding with the exponential growth of UK Universities and pre-registration occupational therapy (OT) education’s transition from Diploma to Bachelor of Science (BSc Hons) degree entry-level qualification (Royal College of Occupational Therapists, 2019a; Ryan, 2001). These educational reforms challenged selection and recruitment processes that had historically seen low income families, males and mature married females excluded from OT; reinforcing the image it was for White, middle-class females (Yates, 1996). Despite a long and clear history of debate, calls for improvement and UK governmental plans to improve access and attainment gaps, certain student groups across socioeconomics, geography, disability and ethnicity, continue to be underrepresented in HE (McLellan et al., 2016; Office for Students, 2019). Similarly, over several decades, the OT community has called for action, to diversify student and workforce populations that mirror the profession’s increasingly diverse service user populations (Atwal, 2021; Colaianni et al., 2022; Ford et al., 2021; Taylor, 2007). Yet, contemporary data suggests a continued lack of diversity within OT. While just 17.5% (*n* = 7,175) of a possible 41,000 registered occupational therapists offered demographic details, the workforce appears to be dominated by those who identify as White (87%), heterosexual (90%), able-bodied (88%) women (92%) (Health and Care Professions Council (HCPC), 2021).

Students undertaking UK pre-registration OT training enter via undergraduate (BSc (Hons), Master of Science (MSc) and degree-level apprenticeship) or postgraduate (MSc and Post Graduate Diploma (PGDip) education pathways. Completion via either pathway requires graduates to meet the UK HCPC standards of education and training, leading to eligibility for registration and practice as a UK occupational therapist (HCPC, 2017). Selection and recruitment processes must include appropriate academic and professional entry criteria (HCPC, 2017; Royal College of Occupational Therapists, 2019b), although neither the Royal College of Occupational Therapists (RCOT) or HCPC offer specific guidance on how these skills should be identified, assessed or recorded. If the profession hopes to diversify workforce populations, it must prioritise diversifying student populations. A key first step lies in identifying and exploring UK Universities’ admissions processes, ensuring they are fair, accessible and transparent, with reliable selection assessment methods used and inequalities professionally addressed, tackled and embedded in governance (Universities UK, 2020). It is difficult to understand, with no published investigation of UK OT entry criteria, if there is equity and accessibility; for candidates to appreciate what is required; and for the profession to know who is missing or excluded at the point of enquiry and entry. Using a cross-sectional design, this study aims to address this gap by providing, for the first time, an overview of current UK admissions criteria. Visiting all UK university websites offering pre-registration OT education, it records available programme types, while exploring what and how academic, professional and alternative entry criteria are assessed at the point of application and selection.

## Literature review

Research specific to OT recruitment spans several decades, having gained increased momentum in recent years. The literature review explores contemporary international evidence on the academic, professional and widening participation entry criteria utilised by universities.

## Academic (cognitive) entry criteria

Evidence to support the use of cognitive data as a reliable predictor of graduate success is variable across both UK and United States (US) contexts. First-time pass rates for the National Board for Certification in Occupational Therapy (NBCOT) examination were compared with pre-admission factors and academic programme performance for 144 MSc students at a single US university (Novalis, Cyranowski and Dolhi, 2017). Identifying an 81.94% pass-rate (*n* = 118) and an 18.06% fail-rate (*n* = 26) at first attempt, those who failed were found to have a lower in-programme Grade Point Average (GPA), been placed on a modified academic programme and more likely to be male. Pre-admission GPA scores did not correlate with examination success or failure, but those who failed, scored lower on pre-admission writing sample scores (Novalis, Cyranowski and Dolhi, 2017). A review of academic admissions requirements revealed 99.4% MSc (*n* = 155) and 100% OT Doctorate (OTD, *n* = 16) American Occupational Therapy Association (AOTA) accredited programmes required evidence of minimum pre-admission GPA scores. Submission of at least one Graduate Record Examination (standardised test used as an admissions requirement for many North American graduate schools) score was also required by 53.9% (*n* = 84) of MSc and 68.8% (*n* = 11) of OTD programmes (Bowyer et al., 2018). McGinley (2020) compared pre-admission undergraduate qualifications of one cohort of UK pre-registration BSc students (*n* = 44), with final degree outcomes, finding no relationships. Three candidates entered under minimum academic requirements and achieved First Class (*n* = 1) and Second Class (*n* = 2) degrees. Strict exclusion criteria including programme withdrawals, late graduations and prior degree-level qualifications resulted in a final sample (*n* = 27), affecting generalisability. McNeil et al. (2021) found no statistical difference between 111 MSc and OTD programmes (from the top 107 US health schools) who achieved 100% versus less than 100% NBCOT pass rates and academic entry criteria. However, detail of the methodology used to rank the programmes was limited and may have skewed results (McNeil et al., 2021). Limitations aside, both studies agree final academic attainment is not directly correlated with academic entry criteria, highlighting the need for further research to explore variables beyond those collected at admission (McGinley, 2020; McNeil et al., 2021).

## Professional (non-cognitive) entry criteria

A 36-item survey of AOTA BSc programmes (*n* = 73) explored the content, purpose and effectiveness of admissions interviews. With a response rate of 68% (*n* = 50), results showed that 48% (*n* = 24) used interviews as a selection tool (Agho et al., 1998). Two decades later, a survey of AOTA programmes offering MSc and OTD level OT education (*n* = 155) yielded a 20% (*n* = 31) response rate. Evidence revealed 66.7% (*n* = 20) used interviews, comprising single applicant and panel (*n* = 8); multiple applicants and panel (*n* = 2); single applicant/single interviewer (*n* = 6) or multi-mini interviews (MMIs; *n* = 4) (Bowyer et al., 2018). In an attempt to offer reliable evidence-based alternatives to traditional interviews, the behavioural interview (BI) and MMI have been identified and explored (Bowyer et al., 2018; Kale et al., 2020; Li et al., 2017). They have received positive attention for evaluating non-cognitive capacity through values-based scenarios, while tackling issues relating to labour, time and cost-intensity, bias, validity and reliability but correlational evidence between admission and graduation success is variable (Eva et al., 2004; Grice, 2014). Despite inconsistent supportive evidence, interviews continue to remain popular, with 61.26% of MSc and OTD programmes (*n* = 111) in the US using them, alongside letters of recommendation (97.30%), personal statements (90.09%) and observation hours (74.77%) (McNeil et al., 2021). The study found no statistically significant differences in non-cognitive admissions criteria between those programmes that gained a 100% pass rate at NBCOT compared to those who did not across all four themes. Furthermore, comparing cumulative scores of a reflective essay and psychometric test with final degree outcomes found no significant correlations in either direction for one cohort of UK BSc (Hons) OT students (*n* = 27; McGinley, 2020).

## Widening participation (alternative) entry criteria

OT-specific widening access and participation literature focuses on the lived experiences of students entering the profession with ‘non-traditional’ academic qualifications (access and vocational qualifications, foundation degrees and/or Advanced Levels (A-Levels) achieved as a mature student) from a range of backgrounds spanning age, gender, ethnicity and socioeconomics (Greenwood et al., 2007; Ryan, 2001; Watson, 2013). With the exception of one national US survey, showing age, marital status and ethnicity not to be predictors of first-time success at the NBCOT exam (Novalis, Cyranowski and Dolhi, 2017), the limited evidence is restricted to small-scale or single-site studies. Ryan (2001) adopted a qualitative approach to explore the narratives of five mature OT students, uncovering critical educational incidents with specific relevance for admissions tutors seeking to widen access. Examples include the need for suspension of minimum entry requirements in recognition of prior experience(s); bespoke mentorship to ensure not only access to, but smooth transition and continuation of, education and greater programme flexibility, such as part-time opportunities. Focusing on UK students (*n* = 194) enrolled on science-based Access to Higher Education (Access to HE) programmes, Greenwood et al. (2007) explored familiarity with six Allied Health Professional careers (including OT), assessing whether providing a small amount of information about profession(s) would impact a student’s decision to consider it as a future career. Statistical significance was found for those students who ‘knew nothing’ about OT (*n* = 27), with 10 reporting they would consider training after reading information about the profession. While a single, aged study, evidence suggests that targeting access to HE students with information about OT could widen access and participation to the profession. Watson (2013) considered the influence of UK BSc (Hons) OT students’ (*n* = 239) background characteristics, finding no correlation between pre-admission qualifications, age, gender or socioeconomic background and final degree outcomes. However, being male and/or from a lower socioeconomic background were significant predictors of poorer outcomes for programme progression. This study offers a unique perspective on participation and progression once access has been widened, highlighting the need to ensure students from diverse backgrounds receive sufficient support to proceed and succeed at all levels.

## Method

### Sample

The RCOT Career Handbook (Royal College of Occupational Therapists, 2020, 2021) was used to identify the sample and guide inclusion and exclusion criteria of the final sample size of UK Universities (*n* = 40), offering RCOT accredited pre-registration OT education programmes (*n* = 73) for entry in the academic year of 2021/2022 (Table 1).

**Table 1.** Inclusion and exclusion criteria.

Inclusion criteria	Exclusion criteria
UK Universities in England, Ireland, Scotland and Wales.	Universities based outside of the UK.
OT programmes, accredited by the RCOT to provide pre-registration OT education.	Pre-registration OT programmes awaiting professional RCOT accreditation.
Full-time/part-time pre-registration OT programmes across undergraduate and postgraduate routes.	Post-registration OT (MSc, PGDip, Doctoral) programmes for registered /qualified Occupational Therapists seeking postgraduate options.

### Design

A cross-sectional research design was used, focusing on collection of quantitative data from public websites and subsequent content analysis. This non-reactive method allowed for exploration of information to identify and describe potentially emerging themes from the extracted data (Neuman, 2011).

### Ethics

Institutional ethical approval was gained. Informed consent was not required due to the public nature of the data explored.

### Data collection and analysis

Between October 2020 and March 2021, websites of all UK Universities offering pre-registration OT programmes were explored. In line with ethical considerations, once data collection was complete, each university was allocated a number (1–40) and data was cleaned of identifiable references. Specified entry criteria for each individual programme, across academic (cognitive), professional (non-cognitive) skills and alternative (widening participation) categories were recorded into a single Microsoft Excel™ document. A coding schedule was developed against a pre-determined set of variables with coded data entered into Statistical Packages for Social Sciences for descriptive analysis. (Bryman, 2016; IBM Corp., 2020; Table 2).

**Table 2.** Variable names and categories.

Variable name	Variable categories
Programme	Type (BSc (Hons), MSc (UGr), Apprenticeship, MSc (PGr), PGDip)
	Length (2, 3, 4, 4–6 years)
	Attendance (full-time/part-time)
Academic (cognitive) entry criteria	Level 2 qualifications (General Certificate of Education (GCSE) subject(s), number and grades; acceptance of Level 2 alternatives, excluded subjects)
	Level 3 qualifications (A-Level, Access to Higher Education (HE), British and Technology Education Council (BTEC), Scottish Highers, Welsh Baccalaureate and Irish Leaving Certificate), subject(s), grades, UCAS tariffs* across Level 3 options, excluded subjects)
	Level 5 qualifications (foundation degree type, subject(s), grades)
	Level 6 qualifications (undergraduate degree type, subject(s), grade, time limits, accepted alternatives
Professional (non-cognitive) entry criteria	Are professional skills assessed at point of **application**? (Yes/No – If yes, how is this evidenced?)
	Are professional skills assessed at point of **selection**? (Yes/No – If yes, how is this evidenced?)
Alternative entry criteria	Alternative entry routes offered? (Yes/No – If yes, what are they?)
	Covid-19 changes to selection & recruitment? (Yes/No – If yes, what are they?

* Universities and Colleges Admission Service (UCAS) Tariff tables.

Access to the raw and coded data is available via the Open Science Framework (https://osf.io/m4jhw/?view_only=1e2fa3e7a86e4463bae563c8d96fed62)

As there is no standardisation to the advertising of UK Level 3 qualifications, UCAS Tariff tables (UCAS, 2021; the translation of qualifications and grades into a numerical value for the purposes of course entry requirements) were used to convert these qualifications into an assigned numerical tariff so that data analysis could be performed.

## Findings

### Programme type

Forty UK universities deliver 73 RCOT accredited pre-registration OT programmes of education. These are split between three undergraduate routes consisting of BSc (Hons) referred to hereafter as BSc (49%, *n* = 36); MSc (UGr) 3%, *n* = 2); apprenticeships (11%, *n* = 8) and two postgraduate routes consisting of MSc (PGr) 34%, *n* = 25); PGDip (3%, *n* = 2), which equate to a broader spread of total undergraduate (63%, *n* = 46) and postgraduate (37%, *n* = 27) options. Applicants have a choice of full-time (F/T, 85%, *n* = 62) and part-time (P/T, 15%, *n* = 11) programmes. While the eight apprenticeships are advertised as either 4 years P/T (*n* = 3); 3 years P/T (*n* = 4) or 3 years F/T (*n* = 1), they all require applicants to be working in close regular contact with an occupational therapist(s) in addition to the academic programme. The remaining four P/T options are split between BSc (*n* = 3) and MSc (PGr) (*n* = 1).

### Academic (cognitive) skills entry criteria

#### Level 2 requirements (GCSE or equivalents)

The number of required GCSEs are not stipulated by the majority of postgraduate (81%, *n* = 22) and half of undergraduate (50%, *n* = 23) programmes. When stipulated, the minimum number of GCSEs required ranges from three to five subjects, with almost one-third of all programmes requiring five subjects (30%, *n* = 22) and over half not stating a minimum number (62%, *n* = 45). A popular GCSE grade requirement is grade 4/C as a standard pass (71%, *n* = 52), with the remainder of programmes either not explicitly stating a minimum grade (25%, *n* = 18) or requiring a grade 5/B and above in English Language, Maths and Science (4%, *n* = 3). Specifically focusing on undergraduate programmes, Grade 4/C is a popular requirement for English (96% *n* = 44), Maths (91% (*n* = 42) and Science (46%, *n* = 21). If a candidate does not have GCSE qualifications in Maths and/or English, some universities permit ‘Functional Skills’ as an alternative, designed as a qualification for ‘*work, study and life*’ (Department for Education, 2018, p. 4). Over half of all programmes do not explicitly state their position in relation to Functional Skills (67%, n = 49). By contrast, just over a quarter demonstrate visible acceptance (26% *n* = 19) or rejection (7%, *n* = 5) of these alternative qualifications through programme websites.

#### Level 3 requirements (A-Levels)

As postgraduate programmes do not publicise Level 3 academic qualifications at the point of entry, this section reports wholly on undergraduate programmes (*n* = 46). Of these, four (BSc, *n* = 1; apprenticeship, *n* = 3) do not stipulate A-Level grades or equivalent UCAS tariff points for entry. Minimum UCAS tariff points across BSc and MSc (UGr) (*n* = 37) range from 96 to 128 (equivalent to A-Level grades CCC – ABB), with apprenticeships (*n* = 5) ranging from 104 to 128 (equivalent to BCC – ABB). Eighteen (39%) BSc programmes require minimum entry of 120 tariff points at A-Level (equivalent to BBB), with twelve across both BSc and apprenticeship requiring a minimum tariff of 112 (equivalent to BBC). Students not achieving this minimum, face few access options, with less than 20% (*n* = 9) offering a tariff below 112 points. 39% (*n* = 18) of programmes identify required A-Level subjects as a Science (pure, social or Physical Education (PE), *n* = 15), English (*n* = 1) or no preferred subjects (*n* = 2). However, consistent data is limited, with 61% (*n* = 28) having no visible subject requirement. A-Level General Studies is excluded (35%, *n* = 16), with no clear or direct explanation.

#### Level 3 requirements (Access to HE, BTEC, Scottish Highers, Irish Leaving Certificate, Welsh and International Baccalaureates)

39% (*n* = 18) of undergraduate programmes do not stipulate required entry grades or UCAS tariff equivalents for Access to HE qualifications. Tariff point offers range from 64 to 128 (equivalent to A-Level grades CC – ABB). The most commonly occurring minimum tariff occurs at 122 points (*n* = 7) and although not easily equated to an A-Level equivalent due to a ±2-point discrepancy, the closest is BBB. BTECs are cited as an entry option for some programmes but data is limited, and visibility reduces across each qualification, with 33% (*n* = 15), 80% (*n* = 37) and 93% (*n* = 43) not stipulating entry criteria across the Extended Diploma, Diploma and Extended Certificate/Subsidiary Diploma respectively. Where they are present, minimum tariff point offers range from 104 to 144 (equivalent to BCC – AAA), with the most commonly occurring minimum tariff (*n* = 21) occurring at 128 points (equivalent to ABB). Data for the devolved nations and European qualifications is limited, with between just two and fourteen programmes stipulating minimum entry criteria for each qualification. A range of minimum entry criteria is seen, with the lowest minimum tariff of 80 points (equivalent to CCE) stated for the European Baccalaureate and the highest minimum tariff of 147 (closest equivalent of 144 points, equivalent to AAA) for Scottish Highers.

#### Level 5 requirements (foundation degree)

17% (*n* = 8) undergraduate programmes clarify whether Health and Science Foundation Degrees (H&SFD) are accepted for entry. Of these, six require candidates to have undertaken their own institutions’ FD, with two accepting a H&SFD from any institution.

#### Level 6 requirements (undergraduate degree)

As most undergraduate candidates would not be expected to have achieved a previous degree, this section focuses wholly on postgraduate programmes (*n* = 27). All require evidence of a previous degree, with a near equal split requiring either a 2:1 (48%, *n* = 13) or 2:2 (52%, *n* = 14). Fifteen programmes (56%) do not specify an imposed time limit on achievement of a previous degree prior to application and all that do are MSc (6 years, *n* = 2); 5 years, *n* = 8; 3 years, *n* = 2). Subject requirements include a Science (pure or social, *n* = 12), not explicitly stated (*n* = 10) or any subject (*n* = 5). Few programmes stipulate acceptable alternatives to a classified degree, with 78% (*n* = 21) not making it clear whether alternative entry equivalencies would be considered. Of the six programmes that do offer an alternative route, all are MSc. Accepted alternative entry routes relate to acceptance of a 2:2 over a 2:1; previous degree negated OR non-science degree where relevant work experience is evident, a professional qualification (such as Teaching or Social Work) and/or relevant Level 3 qualifications. One programme offers a pre-entry online research methods module for those who have (a) not recently studied, (b) a 2:2 or third-class degree, (c) relevant work experience or (d) a gap in research knowledge. It is assumed this is an in-house programme.

### Professional (non-cognitive) skills entry criteria

Three-quarters of all programmes stipulate the requirement for evidence of professional skills at the point of application (77%, *n* = 56). Of the 23% (*n* = 17) that do not specify this, 15% (*n* = 11) are undergraduate and 8% (*n* = 6) postgraduate. Although the UK UCAS system requires all candidates to submit a personal statement at the point of application, just under one-third of programmes state this requirement on websites (32%, *n* = 23). As highlighted in Table 3, six further entry criteria themes were identified in the data, with 58% (*n* = 42) of all programmes stating assessment of two or more themes at the point of application. Furthermore, the majority make explicit reference to professional skills assessment at the point of selection (82%, *n* = 60), with the most popular methods being interviews (*n* = 63), including interview (*n* = 58), group interview (*n* = 3) and MMIs (*n* = 2); values-based assessment (*n* = 19) and group work (*n* = 11; Table 3).

**Table 3.** Professional skills assessed at the point of application and selection by UK pre-registration OT programmes (*n* = 73).

	Number of programmes (%)	
Professional skills at application		
Not Stipulated	17 (23)	
Work Experience	38 (52)	
Knowledge of profession/role and/or scope of practice	36 (50)	
Individual personal qualities	25 (34)	
Personal Statement	23 (32)	
Values-based assessment/NHS Values/NHS Constitution	19 (26)	
Personal Experience	9 (12)	
Professional skills at selection		
Not Stipulated	13 (18)	
Traditional Interview	58 (80)	} Total: 63 (87%)
Group Interview	3 (4)	
Multiple-Mini Interview	2 (3)	
Values-Based Assessment	19 (26)	
Group Work	11 (15)	
Written Component	7 (10)	
Work Experience	6 (8)	
Selection Days	3 (4)	
Service User Advocacy Task	2 (3)	
Portfolio Review	1 (1)	

At the time of data collection, the UK entered two national lockdowns as a result of Covid-19 (Institute for Government Analysis, 2021), posing restrictions on travel and a requirement to work from home. Websites were explored for changes to the 2021/2022 selection and recruitment process, with 8% (*n* = 6) of programmes referring to any changes relating to face-to-face (F2F) interviews replaced by telephone calls (*n* = 2), online interviews (*n* = 2) and relaxation of formal work experience requirements (*n* = 2).

### Alternative entry criteria

67% (*n* = 49) of all pre-registration programmes stipulate consideration of alternative entry routes. Information for candidates is variable between institutions, with the most commonly occurring themes relating to Recognition of Prior Learning (RPL; *n* = 20); work experience (*n* = 17); case by case (*n* = 17); mature student (*n* = 10) and life experience (*n* = 7). While these themes may be considered as widening access, very few institutions explicitly use this language as a theme (*n* = 4). Of the 49 programmes that stipulate alternative entry criteria, 55% (*n* = 27) state singular options, with 45% (*n* = 22) offering two or more possible alternative entry routes (Table 4).

**Table 4.** Alternative OT entry routes considered by UK university pre-registration programmes (*n* = 73).

Alternative route	Total number of programmes (%)	Number of programmes offering as a single approach (%)
Not Stipulated	24 (33)	24 (33)
Recognition of Prior Learning (RPL)	20 (27)	8 (11)
Work Experience	17 (23)	2 (3)
Case by Case	17 (23)	11 (15)
Mature Student	10 (14)	2 (3)
Life Experience	7 (10)	1 (1)
Widening Access	4 (6)	1 (1)
Foundation Degree (own institution)	4 (6)	2 (3)
Level 3 Apprenticeship	1 (1)	0 (0)

## Discussion and implications

Results from this exploratory research suggest candidates face a lack of parity and transparency of academic skills; use of professional skills assessment tools, backed by variable evidence; and a range of alternate but inconsistent routes into the profession. Coupled with a deficiency in part-time opportunities, these factors have the potential to influence diverse student and workforce populations, as discussed in more detail below.

### Programme type

Eleven programmes are advertised as P/T, seven being apprenticeship, requiring close and regular contact with an occupational therapist(s). This option is therefore only open to candidates who have secured employment with a service willing to support their education and who are able to commit to a combination of employment and study. This leaves four programmes offering a P/T route via one MSc and three BSc. With 62% of F/T and 100% of P/T students aged 21 years and older (Royal College of Occupational Therapists, 2017), mature students are clearly attracted to and accessing the profession. These students are likely to arrive with a non-traditional academic background as well as a wealth of life, work and prior learning experience (Watson, 2013). However, with so few P/T programmes available nationally, is it possible the profession is limiting or preventing access to those who have caring, financial or other life commitments, while favouring those with privileged access to financial, physical, emotional, social and cultural support? As highlighted by Watson (2013), there is an ‘uncomfortable paradox’ in valuing and supporting diversity in OT service user populations, while not doing the same with and for student populations (p.526).

### Academic entry criteria

Despite clear gaps in the visibility of academic entry criteria across all programmes, there are some consistencies. Data suggests an applicant is more likely to find clear and consistent academic entry criteria if they have taken or are taking ‘traditional’ qualifications; namely GCSEs, A-Levels and/or a BSc degree. However, equity between different Level 3 academic qualifications is variable, as evidenced when minimum ranges of tariff points are compared between A-Levels (96 – 128 tariff points); Access to HE (64–128 tariff points) and BTEC (104–144 tariff points). This suggests BTEC students are expected to perform higher than A-Level students and further still to Access to HE students. Even so, caution must be exercised due to the varying number of programmes that do not publish entry criteria for Access to HE (*n* = 18) or BTEC (Extended Diploma, *n* = 15; Diploma, *n* = 37; Extended Certificate/Subsidiary Diploma, *n* = 43), compared to A-Levels (*n* = 4). Furthermore, not all tariff point options for Access to HE, BTEC, Scottish, Irish, Welsh and European equivalents are easily transferrable or comparable to A-Level tariff equivalencies. As there is such limited data available (A-Levels aside), candidates looking to enter with an alternative Level 3 qualification may find the system difficult to navigate. Without further investigation and effort from the candidate, these combined factors highlight a system that lacks demonstrable fairness across qualifications that carry the same credits.

### Professional entry criteria

This research found professional skills were explicitly considered and promoted to candidates across at least three-quarters of all UK programmes via institutional websites. Findings support the international OT evidence, which collectively demonstrate work experience, knowledge of the profession, written personal statements and performance at interviews as popular decision-making selection tools (Agho et al., 1998; Bowyer et al., 2018; McNeil et al., 2021). Additionally, entry requirements related to evidence of personal qualities and values; awareness of the NHS Constitution; and/or a lived experience of OT practice are highlighted, although it is not obvious as to how personal statements are scrutinised for progression beyond application. In order for candidates to evidence these qualities, they need to have had access and exposure to the OT profession, something considered to aid academic and professional success, but not backed by evidence (McNeil et al., 2021). Covid-19 will undoubtedly have had an impact on candidates’ ability to gain direct work experience, affecting their demonstrable commitment to, and understanding of, the profession at application and selection. With so many programmes requiring evidence of work experience, it is curious to observe just two have relaxed this requirement. This raises further questions regarding who may be excluded or missed because of a lack of evidenced exposure.

Despite a lack of evidence to support traditional interviews, they have historically been used as a tool to determine entry into OT education (Bowyer et al., 2018; Grice, 2014). Results reflect this trend, with 80% (*n* = 58) of UK programmes choosing to utilise interviews. Alternatives such as the MMI and BI have identified encouraging benefits for interviewers and interviewees, as well as variable correlations to graduate outcomes (Grice, 2014; McNeil et al., 2021). Even so, the use of MMIs and group interviews appear not to be commonplace in the UK, with 3% (*n* = 2 MMIs) and 4% (*n* = 3 group interviews) of programmes detailing these as part of their selection process. With a clear need to assess professional skills at selection, this adds weight to the call for OT admissions tutors to consider MMIs and BIs as a potential part of admissions criteria (Bowyer et al., 2018; McGinley, 2020). This is especially important given the considered value of the MMI in offering assessment of values-based scenarios (Eva et al., 2004), which may be a useful alternative for those candidates who cannot evidence direct exposure following Covid-19.

### Alternative entry criteria

Over two-thirds of all programmes (67%) acknowledge potential alternative routes into the profession, with 24 programmes (33%) showing no visible consideration of how students with a non-traditional academic background will be considered at admission. Research suggests if people do not see people like themselves in a profession, they will not pursue it as a career (Ford et al., 2021). Given the issues related to stagnant progress in increasing diversity that reflect the communities’ occupational therapists’ work alongside (Atwal, 2021; Taff and Blash, 2017; Taylor, 2007; Yates, 1996), this is an important national and international consideration for OT education providers to consider when publicising alternative entry criteria at the point of enquiry.

### Limitations

This research demonstrates an apparent lack of standardised entry criteria, confirming the existence of bespoke but inconsistent selection processes between UK universities. While this is the result of each institution offering individual OT programmes as opposed to a single homogenous organisation, this poses challenges for navigators of advertised entry criteria. Namely, the profession, institutions, admissions tutors and pre-registration candidates alike. It was beyond the scope of this unobtrusive research to seek the perspectives, experiences and narratives of these stakeholders and is therefore a significant limitation of this exploratory and preliminary dataset. Furthermore, with data collected over a 6-month period, there may have been intermittent or ongoing changes in the data the researchers were unaware of.

### Further areas of work

This research highlights challenges for potential OT candidates in relation to what academic qualifications to choose, as well as grade(s) to achieve, work experiences to gain and how lived experiences may be considered as an alternative to traditional academic and professional entry routes. Additionally, a lack of P/T programmes have the potential to limit access for those who have caring, financial or other life commitments. Priority areas for future research should therefore include exploration of the barriers to access and participation as experienced by those who have not taken a traditional academic or professional route and for whom F/T study may not be an option. With continuing concerns over a lack of diversity within the profession, understanding candidates’ lived experiences of selection and recruitment may assist in developing an evidence-base that aims to appreciate if current UK admissions processes are influencing OT student and eventual workforce demographics.

## Conclusion

This study has detailed current UK pre-registration OT education entry criteria through the systematic identification and recording of data via university programme websites. It has explored issues related to equitable and accessible processes, detailed candidate expectations and debated who may be missing or excluded at the point of enquiry and entry. It has identified a paucity of P/T courses, a focus on traditional entry routes and assessment by interview, with limited information on alternative entry criteria. With ongoing calls to actively diversify student and workforce populations, a reliance on traditional academic and professional entry criteria has the potential to reinforce a lack of student diversity. If the profession is to break this cycle, it is essential UK universities increase parity across academic entry criteria, ensure the visibility of acceptable skills for alternative access and substantially improve flexibility for P/T study. Fair and transparent admissions processes that aim to widen access and participation have the potential to serve as an initial step in ensuring the OT profession benefits from a diverse studentship and eventual workforce.

**Key findings**

- There is variable visibility, transparency and parity in advertised UK pre-registration OT entry criteria.
- Current systems have the potential to reinforce a continued lack of diversity in the profession.

**What the study has added**

If the profession is to diversify, there is an urgent need for UK universities to ensure parity across entry criteria, increase the visibility of acceptable alternative routes and substantially improve flexibility for part-time study.

## Supplemental Material

sj-docx-1-bjo-10.1177_03080226221148412 – Supplemental material for Selection and recruitment of pre-registration occupational therapy students in the United Kingdom: Exploring entry criteria across education providers Supplemental material, sj-docx-1-bjo-10.1177_03080226221148412 for Selection and recruitment of pre-registration occupational therapy students in the United Kingdom: Exploring entry criteria across education providers by Sarah Louise McGinley, Sukhbinder Hamilton and Alexander Bradley in British Journal of Occupational Therapy

sj-sav-2-bjo-10.1177_03080226221148412 – Supplemental material for Selection and recruitment of pre-registration occupational therapy students in the United Kingdom: Exploring entry criteria across education providers Supplemental material, sj-sav-2-bjo-10.1177_03080226221148412 for Selection and recruitment of pre-registration occupational therapy students in the United Kingdom: Exploring entry criteria across education providers by Sarah Louise McGinley, Sukhbinder Hamilton and Alexander Bradley in British Journal of Occupational Therapy This article is distributed under the terms of the Creative Commons Attribution 4.0 License ( http://www.creativecommons.org/licenses/by/4.0/) which permits any use, reproduction and distribution of the work without further permission provided the original work is attributed as specified on the SAGE and Open Access pages ( https://us.sagepub.com/en-us/nam/open-access-at-sage).

sj-xlsx-3-bjo-10.1177_03080226221148412 – Supplemental material for Selection and recruitment of pre-registration occupational therapy students in the United Kingdom: Exploring entry criteria across education providers Supplemental material, sj-xlsx-3-bjo-10.1177_03080226221148412 for Selection and recruitment of pre-registration occupational therapy students in the United Kingdom: Exploring entry criteria across education providers by Sarah Louise McGinley, Sukhbinder Hamilton and Alexander Bradley in British Journal of Occupational Therapy This article is distributed under the terms of the Creative Commons Attribution 4.0 License ( http://www.creativecommons.org/licenses/by/4.0/) which permits any use, reproduction and distribution of the work without further permission provided the original work is attributed as specified on the SAGE and Open Access pages ( https://us.sagepub.com/en-us/nam/open-access-at-sage).
