# Supplementary material for: Selection and recruitment of pre-registration occupational therapy students in the United Kingdom: Exploring entry criteria across education providers
Source: Br J Occup Ther. 2023 Jan 11;86(5):385–93. doi: 10.1177/03080226221148412 (PMC12033753; doi:10.1177/03080226221148412)
Supplement: sj-docx-1-bjo-10.1177_03080226221148412 – Supplemental material for Selection and recruitment of pre-registration occupational therapy students in the United Kingdom: Exploring entry criteria across education providers [file sj-docx-1-bjo-10.1177_03080226221148412.docx]

**Phase 1: Content Analysis Coding Manual – Quantitative Data**

| **Variable Name (Label in SPSS)** | **Value Options** | **Type of Measure** |
| --- | --- | --- |
| 1. University Code (Identifier) | Numbered 1, 2, 3, etc until 40 | Nominal |
| 1. University Type (All Types) – for descriptive statistics | 1 = Russell Group University  2 = 1960’s (Plate Glass University)  3 = Post 1992 (New University) | Nominal |
| 1. University Type – for inferential statistics | 1 = Pre-1992  2 = Post-1992 |  |
| 1. Programme Type - for descriptive statistics | 1 = BSc  2 = MSc (PGr)  3 = PGDip  4 = Apprenticeship  5 = MSc (UGr) | Nominal |
| 1. Programme Type – for inferential statistics | 1 = Undergraduate (BSc, MSc & Apprenticeships)  2 = Postgraduate (MSc & PGDip) | Nominal |
| 1. Programme Length | 2 = 2 years  3 = 3 years  4 = 4 years  5 = 4-6 years | Nominal |
| 1. FT/PT Attendance | 1 = Full-time  2 = Part-time | Nominal |
| 1. # of GCSEs required (Level 2) | 0 = Not stipulated  3 = 3 GCSEs  4 = 4 GCSEs  5 = 5 GCSEs | Ordinal |
| 1. Minimum grade GCSEs (Level 2) | 0 = Not stipulated  4 = Grade 4/C – Standard Pass  5 = Grade 5/C – Good Pass  6 = Grade 6/B | Ordinal |
| 1. GCSE English requirement (Level 2) | 0 = Not stipulated  1 = English Language  2 = Literature AND Language | Nominal |
| 1. GCSE Maths requirement (Level 2) | 0 = Not stipulated  1 = Yes | Nominal |
| 1. GCSE Science requirement (Level 2) | 0 = Not stipulated  1 = Yes | Nominal |
| 1. Level 2 Alternatives Accepted? (Level 2) | 0 = Not stipulated  1 = Yes  2 = No | Nominal |
| 1. A Level UCAS Tariff Scores (Static) (Level 3) | 0 = Not stipulated  1 = Tariff range offered  96 = 96 tariff points  104 = 104 tariff points  112 = 112 tariff points  120 = 120 tariff points  128 = 128 tariff points | Ordinal |
| 1. A Level UCAS Tariff Scores (Low Range) (Level 3) | 0 = Not stipulated  1 = Static tariff offered  96 = 96 tariff points  104 = 104 tariff points  112 = 112 tariff points  120 = 120 tariff points  128 = 128 tariff points | Ordinal |
| 1. A Level UCAS Tariff Scores (High Range) (Level 3) | 0 = Not stipulated  104 = 104 tariff points  112 = 112 tariff points  120 = 120 tariff points  128 = 128 tariff points  136 = 136 tariff points  144 = 144 tariff points | Ordinal |
| 1. A Level Subject(s) Required (Level 3) | 0 = Not stipulated  1 = No specific subject(s) required by University  2 = Pure Science  3 = Pure OR Social Science  4 = Pure OR Social Science OR PE  5 = English  6 = English AND Pure or Social Science | Nominal |
| 1. Minimum Grade for required A Level Subject(s) (Level 3) | 0 = Not stipulated  1 = Grade B  2 = Grade C | Ordinal |
| 1. A Level Subject(s) Excluded/Not Accepted (Level 3) | 0 = Not stipulated  1 = General Studies Excluded | Nominal |
| 1. Access to HE UCAS Tariff scores (low) (Level 3) | 0 = Not stipulated  64 = 64 tariff points  86 = 86 tariff points  93 = 93 tariff points  96 = 96 tariff points  106 = 106 tariff points  112 = 112 tariff points  115 = 115 tariff points  118 = 118 tariff points  122 = 122 tariff points  128 = 128 tariff points | Ordinal |
| 1. Access to HE UCAS Tariff scores (high) (Level 3) | 0 = Not stipulated  86 = 86 tariff points  93 = 93 tariff points  96 = 96 tariff points  106 = 106 tariff points  112 = 112 tariff points  115 = 115 tariff points  118 = 118 tariff points  122 = 122 tariff points  128 = 128 tariff points  131= 131 tariff points  134 = 134 tariff points | Ordinal |
| 1. BTEC Extended Diploma UCAS Tariff scores (minimum) (Level 3) | 0 = Not stipulated  112 = 112 tariff points  128 = 128 tariff points  144 = 144 tariff points  152 = 152 tariff points  160 = 160 tariff points  168 = 168 tariff points | Ordinal |
| 1. BTEC Diploma UCAS Tariff scores (minimum) (Level 3) | 0 = Not stipulated  104 = 104 tariff points  112 = 112 tariff points  120 = 120 tariff points  128 = 128 tariff points  136 = tariff points | Ordinal |
| 1. BTEC Extended Certificate/Subsidiary Diploma (minimum) (Level 3) | 0 = Not stipulated  96 = 96 tariff points  104 = 104 tariff points  112 = 112 tariff points  120 = 120 tariff points  128 = 128 tariff points | Ordinal |
| 1. Scottish Highers UCAS Tariff scores (minimum) (Level 3) | 0 = Not stipulated  90 = 90 tariff points  112 = 112 tariff points  120 = 120 tariff points  123 = tariff points  128 = tariff points  135 = tariff points  144 = 144 tariff points  147 = tariff points | Ordinal |
| 1. Scottish Highers Advanced UCAS Tariff Scores (Minimum) Level 3 | 0 = Not stipulated  120 = 120 tariff points  128 = 128 tariff points  136 = 136 tariff points | Ordinal |
| 1. Irish Leaving Certificate UCAS Tariff scores (Level 3) | 0 = Not stipulated  102 = 102 tariff points  108 = 108 tariff points  112 = 112 tariff points  114 = 114 tariff points  120 = 120 tariff points  126 = 126 tariff points  128 = 128 tariff points  144 = 144 tariff points  150 = 150 tariff points | Ordinal |
| 1. Welsh Baccalaureate UCAS Tariff scores (Level 3) | 0 = Not stipulated  112 = 112 tariff points  120 = 120 tariff points | Ordinal |
| 1. European Baccalaureate UCAS Tariff scores (Level 3) | 0 = Not stipulated  80 = 80 tariff points  96 = 96 tariff points  104 = 104 tariff points | Ordinal |
| 1. Foundation Degree Accepted? (Level 5) | 0 = Not stipulated  1 = Own University Health & Science FD  2 = Any University Health & Science FD Accepted | Nominal |
| 1. Is Foundation Degree Minimum Grade Stated? (Level 5) | 0 = Not stipulated  1 = Yes  2 = No | Nominal |
| 1. BSc Degree Classification Requirement (Level 6) | 0 = Not stipulated  1 = 2:1 (60-69%  2 = 2:2 (50-59%) | Nominal |
| 1. BSc Degree Subject Required (Level 6) | 0 = Not stipulated  1 = Any subject  2 = Pure OR Social Science | Nominal |
| 1. BSc Degree Time Limits | 0 = Not stipulated  1 = Completed within the last 10 years  2 = Completed within the last 6 years  3 = Completed within the last 5 years  4 = Completed within the last 3 years | Nominal |
| 1. BSc Degree Alternatives Accepted? | 1 = Yes  2 = No specific alternatives stipulated | Nominal |
| 1. Do English Language Requirements meet the HCPC minimum requirement for registration? | 0 = Not stipulated  1 = Yes  2 = No | Nominal |
| 1. Are Alternative Entry Routes Referenced? | 1 = Yes  2 = No | Nominal |
| 1. Professional Skills Assessment –   Is Application Process referenced? | 1 = Yes  2 = No | Nominal |
| 1. Professional Skills Assessment – Is Selection Process referenced? | 1 = Yes  2 = No | Nominal |
| 1. NHS Constitution Referenced? | 1 = Yes  2 = No | Nominal |
| 1. C19 referenced in relation to OT Selection Processes? | 0 = No changes to selection process mentioned  1 = Face to face interviews replaced with telephone interviews  2 = Face to face interviews replaced with virtual online interviews  3 = Direct work experience requirements relaxed | Nominal |

UCAS Tariff Equivalencies taken from 2021 doc - [download (ucas.com)](https://www.ucas.com/file/63536/download?token=sxmdfCS-) (UCAS, 2021)

Reference

UCAS. (2021). *UCAS Tariff tables Tariff points for entry to higher*. https://www.ucas.com/file/63536/download?token=sxmdfCS-
